# Unravelling the Molecular Mechanisms of a Quercetin Nanocrystal for Treating Potential Parkinson’s Disease in a Rotenone Model: Supporting Evidence of Network Pharmacology and In Silico Data Analysis

**DOI:** 10.3390/biomedicines11102756

**Published:** 2023-10-11

**Authors:** Yeruva Sai Lakshmi, D. S. N. B. K. Prasanth, Karumuri Taraka Sunil Kumar, Sheikh F. Ahmad, Seemaladinne Ramanjaneyulu, Nalluri Rahul, Praveen Kumar Pasala

**Affiliations:** 1 Department of Pharmacology, Santhiram College of Pharmacy, JNTUA, Nandyal 518112, Andhra Pradesh, India; sailakshmi27899@gmail.com; 2 Department of Pharmacognosy, KVSR Siddhartha College of Pharmaceutical Sciences, Vijayawada 520010, Andhra Pradesh, India; dsnbkprasanth@gmail.com; 3 Department of Pharmaceutics, Shri Vishu College of Pharmacy, Bhimavaram 534202, Andhra Pradesh, India; sunil123pharma@gmail.com; 4 Department of Pharmacology and Toxicology, College of Pharmacy, King Saud University, Riyadh 11451, Saudi Arabia; 5 Department of Chemistry and Biochemistry, Lamar University, Beaumont, TX 77705, USA; rseemaladinne@gmail.com; 6 Independent Researcher, Kingsville, TX 78363, USA; rnalluri207@gmail.com; 7 Department of Pharmacology, Raghavendra Institute of Pharmaceutical Education and Research, JNTUA, Anantapuramu 515721, Andhra Pradesh, India

**Keywords:** Parkinson’s disease, rotenone, quercetin nanocrystals (QNC), antioxidant system

## Abstract

The prevalence of Parkinson’s disease places a significant burden on society; therefore, there is an urgent need to develop more effective drugs. However, the development of these drugs is both expensive and risky. Quercetin (QUE) has potent pharmacological effects on neurodegenerative diseases, but its low solubility in water and poor bioavailability limit its use in pharmaceutical applications. In this study, Quercetin nanocrystals (QNC) were synthesized and compared to standard QUE. A network-pharmacology-based methodology was applied, including target prediction, network construction, a gene ontology (GO) analysis, a KEGG pathway enrichment analysis, and molecular docking. This study aimed to identify the targets of QUE relevant to the treatment of Parkinson’s disease and investigate the associated pharmacological mechanisms. Most of the predicted targets are involved in dopamine uptake during synaptic transmission. QUE regulates the key targets DRD2 and DRD4, which significantly affect dopaminergic synapses. The molecular docking results showed that QUE had a better binding affinity than the standard drug l-Dopa. From these experiments, it can be concluded that QNC effectively reduced the adverse effects caused by rotenone-induced oxidative stress in biochemical, neurochemical, and histopathological alterations. Therefore, QNC can potentially treat Parkinson’s disease, and its effectiveness should be assessed in future clinical trials.

## 1. Introduction

Parkinson’s disease (PD), a neurodegenerative disorder commonly associated with aging, is caused by the progressive degradation of the dopaminergic neurons in the substantia nigra pars compacta (SNpc). This leads to the appearance of symptoms such as bradykinesia, tremors, and rigidity [1]. Recent research on brains affected by PD has shown that oxidative stress plays a significant role in the development and progression of the disease [2]. This oxidative stress is caused by a disparity between the formation of reactive oxygen species (ROS) and the effectiveness of antioxidant mechanisms, which can potentially damage proteins, lipids, and DNA [3].

Rotenone is a pesticide derived from the roots of plants of the Leguminosae family. The lipophilic nature of the substance allows it to cross both the blood–brain barrier and cell membrane, leading to its neurotoxic properties and the characteristic pathological manifestations of Parkinson’s disease [4]. The primary factor responsible for the neurotoxic effects of rotenone is the generation of oxidative stress, which triggers the manifestation of symptoms that are characteristic of this disease [5]. As a result, rotenone exposure in rats is a valuable experimental model for examining the mechanisms of oxidative-stress-induced dopaminergic damage in Parkinson’s disease.

Quercetin, a naturally occurring flavonoid found in various vegetables and fruits, including tea, apples, mulberries, onions, broccoli, peanuts, and red wine [3], has attracted significant interest from researchers due to its abundance and strong biological activity, which has the potential to prevent health problems such as cardiovascular disease [6], anti-inflammatory effects [7], and neurodegenerative disorders [8]. Furthermore, it has been speculated that Quercetin may function as a unique neuroprotective agent through its ability to scavenge reactive oxygen species [9]. Despite its numerous therapeutic benefits, the pharmacological utilization of Quercetin is limited due to its low solubility and bioavailability [10]. To address these challenges, nanoQuercetin has been employed to optimize the efficacy of therapeutic interventions for a range of disorders, including Parkinson’s disease. Reducing the size of phytomedicines, known as nanosizing, improves their ability to permeate the brain, resulting in an enhanced efficiency and stability [11]. In recent years, nanocrystallization has been proven as effective in facilitating the delivery of medications with a low solubility. The small size and high surface area to volume ratio of nanocrystals have been found to enhance the saturation solubility and dissolution rate of therapeutic particles [12]. Therefore, the primary objective of this study was to evaluate the anti-parkinsonian properties of nanoQuercetin and to compare its efficacy with that of conventional Quercetin in an experimental rat model of Parkinson’s disease.

Network pharmacology is a rapidly advancing field that combines the disciplines of pharmacology, systems biology, and network analysis to study the interactions between biological systems and drugs. Rather than focusing solely on individual drug–target interactions, network pharmacology examines the complex network of molecular components that control the behavior of both biological systems and drugs. This holistic approach allows for a deeper understanding of how drugs function within complex biological networks, which can ultimately lead to the development of more effective and targeted therapeutic interventions [13,14].

To explore the potential treatment options for Parkinson’s disease, we conducted a study that utilized network pharmacology. In this study, we aimed to identify the important targets associated with the treatment of Parkinson’s disease using QUE, evaluate the antiparkinsonian properties of QNC, and compare its effectiveness with that of conventional QUE in an experimental rat model of Parkinson’s disease.

## 2. Material and Methods

### 2.1. Potential Targets Fishing for QUE

The chemical structure of quercetin was obtained from the PubChem database [15] and the target genes were screened from the PharmMapper database (https://www.lilab-ecust.cn/pharmmapper/, accessed on 20 July 2023) [16], Comparative Toxicogenomics Database (https://ctdbase.org/, accessed on 20 July 2023) [17], and Swiss Target Prediction Database (http://www.swisstargetprediction.ch/, accessed on 20 July 2023) [18].

### 2.2. Microarray Data and Differentially Expressed Gene Analysis

The microarray dataset GSE42966 from the Gene Expression Omnibus (GEO) database was collected using the GPL4133 platform (Agilent-014850 Whole Human Genome Microarray 4 × 44 K G4112F) and was downloaded for research purposes [19,20]. This dataset focused on comparing the gene expressions in Parkinson’s disease and normal brain tissue from the substantia nigra region. The aim of the study was to identify the differentially expressed genes (DEGs) in the PD brain, which aligns with the scope of our research. The analysis of differentially expressed genes was conducted using the GEO2R online tool (https://www.ncbi.nlm.nih.gov/geo/geo2r/, accessed on 20 July 2023). Genes with a *p*-value of less than 0.05 and a logarithmic fold change of at least 1.0 were considered to be upregulated, while genes with a *p*-value of less than 0.05 and a logarithmic fold change of less than −1.0 were considered to be downregulated. The results of this analysis are shown in the form of volcano plots. The expression values of the DEGs were obtained using the GEO2R online tool.

### 2.3. Common Targets of QUE and DEG

The QUE and PD treatments share common gene targets. These shared targets were identified and designated as QUE targets in PD treatment.

### 2.4. PPI Network Construction and Hub Gene Identification

We utilized the Search Tool for the Retrieval of Interacting Genes (STRING) database (http://string-db.org/, accessed on 25 July 2023) to examine the protein–protein interaction (PPI) information for previously identified differentially expressed genes (DEGs) [21,22]. To identify possible PPI associations, we employed a cumulative score over 0.7 in the STRING database. Subsequently, the PPI network was visualized using the Cytoscape software (Version 3.10.0) platform (https://www.cytoscape.org/, accessed on 25 July 2023). Nodes with a greater degree of connection played a critical role in maintaining the overall stability of the network. We used the CytoHubba plugin in Cytoscape to determine the degree of each protein node [23]. The top ten genes were identified as hub genes in our study.

### 2.5. GO and KEGG Pathway Analysis of DEGs

A functional enrichment analysis is a common approach used for conducting extensive investigations of gene functions. This method allows for categorizing gene functions into distinct biological processes, molecular functions, and cellular components. The academic community widely uses the KEGG database to store information on genomes, biological pathways, diseases, chemical compounds, and medications. To conduct GO annotation and KEGG pathway enrichment studies on the DEGs, the DAVID tool (https://david.ncifcrf.gov/, accessed on 28 July 2023) was used. The examination of the GO annotations for the DEGs revealed a statistical significance threshold of *p* > 0.05 and a minimum gene count of ≥10 [24].

### 2.6. Molecular Docking

The 2D structure of QUE was obtained from the PubChem database (https://pubchem.ncbi.nlm.nih.gov/, accessed on 30 July 2023) and saved as PDB files for further investigation [25,26]. The structures of the target proteins, Dopamine Receptor D2 (DRD2) (PDB:6CM4) and Dopamine Receptor D4 (DRD4) (PDB:5WIU), were obtained from the Protein Data Bank (PDB) database accessible at http://www.rcsb.org/ (accessed on 30 July 2023) [27]. The protein structures were subjected to hydrogenation, which was performed using PyMOL software (version 2.1.0). The PyMOL plugin was used to explore and visualize the binding pockets. AutoDock Vina [28] was used to hydrogenate each ligand or small molecule, with box dimensions selected to encompass a potential active pocket. Autogrid was employed for the molecular docking simulations. The validation of molecular docking often involves redocking a ligand that is co-crystallized with the protein [29].

### 2.7. QUE Nanocrystal Preparation

The nanocrystals were prepared using a wet media milling technique with a ball mill, and Tween 80 was used as a stabilizer to prevent aggregation. The stabilizer was dissolved in water at 1% *w*/*w*, and the QUE powder was added to the solution and stirred mechanically to ensure adequate wetting. The resulting suspension was introduced into a milling chamber containing zirconium oxide beads, and the milling procedure was conducted at a rotational speed of 300 rpm for four cycles, each consisting of a 5 min milling period followed by a 5 min interval. The nanocrystals were dried and stored in a low-temperature, low-humidity environment until required for subsequent applications. The particle size, zeta potential, and polydispersity index were measured using a Horiba Particle Size Analyzer SZ-100 instrument, and the particle sizes and morphologies of the samples were observed using a scanning electron microscope (JSM–5510-SEM, Make: Jeol, Model: JSM-5510, Tokyo, Japan) [30].

### 2.8. Animals and Ethics Approval

Male Wistar rats weighing 150–180 g were acclimatized for one week. The rats were housed in groups and in propylene cages at a constant temperature of 24 °C, 50–65% relative humidity, and a 12 h/12 h light/dark cycle. The rats received pelleted food and had unrestricted access to water. The study, approved by the Institutional Animal Ethics Committee, utilized 30 Wistar rats (1519/po/Re/S/11/CPCSEA/2022/002).

### 2.9. Chemicals, Software, and Drugs

Rotenone (98%) (Yarrow Chem. Products, Mumbai, India), QUE (95%) (Yarrow Chem. Products, Mumbai, India), DTNB (5,5-dithio-bis-(2-nitrobenzoic acid) (Kemphasol, Mumbai, India), thiobarbituric acid (Loba Chem. Mumbai, India), epinephrine (Chempure, Bangalore, India), trichloroacetic acid (Qualigen, Chennai, India), the PyRx virtual screening tool (BIOVIA Discovery Studio 2017), scanning electron microscopy (JSM–5510-SEM, Jeol Co., Tokyo, Japan), and a Fluorescent Fluorescence Trinocular Microscope (Make: LABOMED, Model: LX 400 TRINO (LED), Supplier—JL Technologies, Hyderabad, India).

### 2.10. Sample Preparation for Animal Study

Rotenone was freshly prepared using dimethyl sulfoxide (DMSO) as a solvent and 10% polyethylene glycol (PEG) at a ratio of 1:1, with a final concentration of 3 mg/kg, as reported by Angeline et al. (2012) [31]. The QUE, QNC, L-DOPA, and QUE suspensions were prepared using Tween 80 and administered orally.

### 2.11. Experimental Procedure

The rats (n = 30) were randomly divided into five groups, each consisting of six animals. Group I was the Negative (DMSO + PEG) Control. Group II was the Positive Control rats treated with rotenone (3 mg/kg body weight I.P) for 21 days. Group III was treated orally with the standard drug L-dopa (10 mg/kg body weight) for 21 days, followed by rotenone (3 mg/kg body weight) administration within a 30 min gap. Groups IV and V were given a Quercetin (QUE) (30 mg/kg body weight) dose chosen from earlier research and QNC (30 mg/kg body weight), respectively, orally for 21 days, before being administered rotenone (3 mg/kg body weight) for 30 min [32]. The motor function of the rats was assessed one day after the end of the therapeutic period (day 21), using various methods to screen their motor functionality.

#### 2.11.1. Pole Test

The pole test apparatus consisted of a wooden pole (50 mm in diameter and 100 cm in height). The rat was placed on top of the wooden pole with their head in the face-up position, and the time taken to reach the floor was measured. The test was repeated thrice for three trials, each lasting 10 min. Behavioral changes were analyzed using the mean of the three descending times [33].

#### 2.11.2. Rotarod Test

The rotarod test was used to evaluate motor coordination and balance. The rats were required to maintain their balance on a revolving rod that was subjected to a rotational speed of 40 rpm. The experiment involved measuring the latency time required for a rat to fall off a revolving rod at 40 rpm [34].

#### 2.11.3. Stair Test

This test was designed to evaluate the level of skilled motor coordination, as outlined by Baird et al. [35]. In this experiment, each rat was positioned at the base of a wooden staircase, inclined at 55 relative to the experimental bench.

#### 2.11.4. Wood-Walking Test

As previously outlined in [36], the wood-walking test was employed to evaluate motor coordination. The rats were permitted to traverse a wooden rod measuring 1 m in length and 5 cm in thickness. The time taken by each rat to reach the terminus of the rod was documented in three consecutive trials per rat.

#### 2.11.5. Wire-Hanging test

The wire-hanging test, often known as the horizontal bar test, was previously introduced [37] to evaluate motor tone. In this experiment, the rats were subjected to a hanging paradigm in which they were suspended from a steel rod by their forelimbs. The rod used in this study had a length of 50 cm and a diameter of 0.8 cm, and was positioned 50 cm above the bench. The duration of latency, which refers to the time that each rat was able to hang from the rod, was measured over three trials. A cutoff time of 60 s was used.

### 2.12. Brain Isolation

The rats were euthanized under ketamine anesthesia (80 mg per kg, i.p.) [38]. After the procedure, their brains were dissected and rinsed with ice-cold phosphate-buffered saline (PBS, pH 7.4). The brain hemispheres were then rapidly frozen at −80 °C for biochemical assays. The process was carried out to ensure the homogenization of the tissue in PBS (10% *w*/*v*), followed by centrifugation and the collection of the supernatant for biochemical assays [39].

### 2.13. Determination of Malondialdehyde and Antioxidant Markers

The supernatants obtained from the homogenization of the rat brains were used to evaluate the malondialdehyde (MDA), reduced glutathione (GSH), catalase (CAT), and superoxide dismutase (SOD) levels. The MDA levels were quantified using a spectrophotometer kit, following the protocol outlined by Ohkawa et al. (1979) [40]. This method relies on the interaction between MDA and thiobarbituric acid, which results in the formation of a pink chromophore. GSH was quantified using Ellman’s reagent, and the absorbance was measured at a wavelength of 412 nm [41]. The activity was determined using the method reported by Aebi (1984) [42], and the absorbance was measured at a wavelength of 240 nm. The CAT activity of catalase (CAT) was evaluated by combining the supernatant (0.5 mL), phosphate buffer (2 mL), and 1 mL of hydrogen peroxide (H_2_O_2_) in a cuvette. The absorbance was determined using spectrophotometry at a wavelength of 240 nm for 30 s. The activity was expressed in terms of the rate of oxidation of H_2_O_2_, measured in nanomoles per minute per mg of protein [43]. The superoxide dismutase (SOD) activity was determined by assessing the extent of the inhibition of nitroblue tetrazolium reduction by superoxide anions [44]. The protein content was measured using the method described by Lowry et al. [45].

### 2.14. Estimation of Dopamine

To prepare the brain tissue homogenates, dopamine and HCl-butanol were used. The resulting reaction mixture was centrifuged at 2000 rpm for 10 min, and a supernatant was collected. The supernatant was then mixed with heptane and hydrochloric acid (0.1 M), and the mixture was centrifuged again at 2000 rpm for 10 min. The aqueous layer was then separated and a small volume was collected. HCl and EDTA were then added to the mixture, and the oxidation process was initiated by adding a small volume of iodine solution. The reaction was stopped by adding sodium sulfate and acetic acid, and the mixture was heated for 6 min at 100 °C. The absorbance of the reaction mixture was then measured at a wavelength of 350 nm. Finally, the dopamine concentration in the brain tissue was estimated using a regression line [46].

### 2.15. Histological Staining

After confirming death by lack of pulse, breathing, corneal reflex, response to firm toe pinch, and heartbeat using a stethoscope, we decapitated and harvested the brain tissues from the rats that were ether anesthetized, and then placed them in 10% formaldehyde for 2 h. The brains were then removed and placed in a new formaldehyde solution for 24 h before being dehydrated in ethanol (70, 90, and 100%) for 24 h. The specimens were then cleaned in xylene and embedded in paraffin. Coronal slices were cut from the brains at a thickness of 5 µm using a Leica RM 2025 microtome, mounted on glass slides, and stained with hematoxylin and eosin [47]. We used Labomed, Inc.’s (Los Angeles, CA, USA) LB-208 Fluorescent Biological Binocular Microscope with an Achromatic Objective to capture the slides and perform morphometric research.

### 2.16. Statistical Analysis

The data are expressed as the mean ± standard error of the mean. To evaluate the statistical significance among multiple groups, a one-way ANOVA, followed by the Dunnet test, was performed using a computer-based fitting program (GraphPad Prism 9.5.3). The predetermined significance level was employed for the analysis [48].

## 3. Results and Discussion

### 3.1. Identification of DEGs

**Figure 1:**
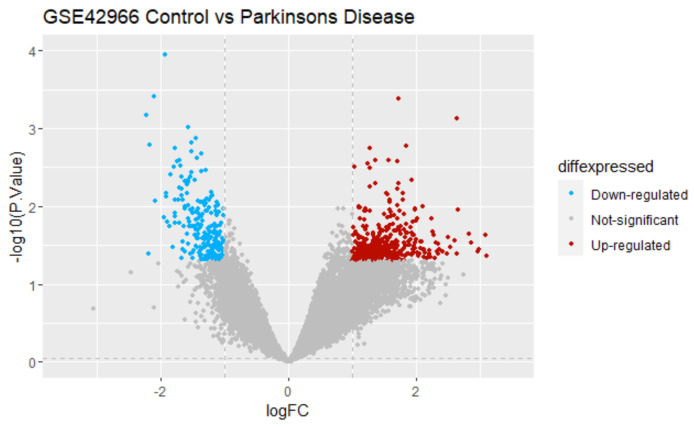
Volcano maps of all DEGs in the GSE42966 dataset. Red: upregulated genes; blue: downregulated genes; and gray: non-significant genes.

The dataset used in this study was GSE42966, which contains nine samples from individuals with Parkinson’s disease and six samples from individuals without the disease, serving as a control. Based on the criteria of log FC ≥ 1.0 and *p* ≤ 0.05, 592 differentially expressed genes (DEGs) were identified from GSE42966. Of these DEGs, 369 were upregulated and 223 were downregulated. The DEGs were identified by comparing the samples from the substantia nigra affected by Parkinson’s disease (PD) with those of the controls. Figure 1 displays a comprehensive volcano map of all the discovered DEGs.

### 3.2. Common Targets for PD and QUE

**Figure 2:**
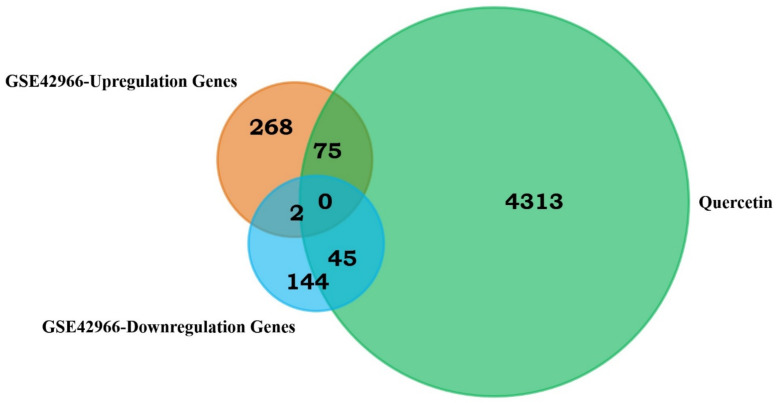
Venn diagram showing the DEGs between Parkinson’s and QUE.

We identified 120 common targets between quercetin and PD using the Venny 2.1 online platform (https://bioinfogp.cnb.csic.es/tools/venny/, accessed on 30 July 2023). In total, 343 genes were upregulated and 189 genes were downregulated in GSE42966 (Figure 2).

### 3.3. PPI Network Construction and Identification of Hub Genes by CytoHubba

**Figure 3:**
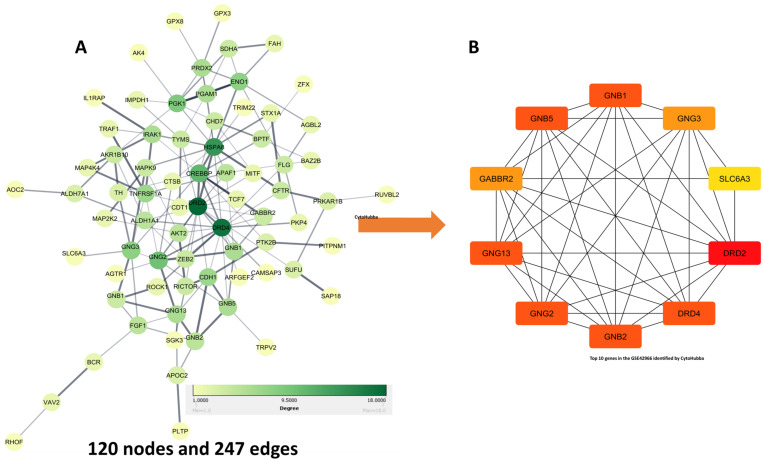
Protein–protein interactions and hub genes. (**A**) Protein–protein interaction (PPI) of target genes. Each node represents all proteins that were common between QUE and GSE42966. Color and size represent the degrees of nodes. (**B**) The Cytohubba plugin was used to select the top ten hub genes. PPI—protein–protein interaction.

The construction of the PPI network involved the importing of 120 targets into STRING (as shown in Figure 3A). This led to the creation of 120 nodes and 247 edges in the network (as depicted in Figure 3). To visualize the obtained PPI network information, the Cytoscape software was used. Using the CytoHubba application and MCC calculation method, the top ten nodes were selected from the network. These nodes were GNG2, GNB2, GABBR2, DRD2, DRD4, GNB5, GNG3, GNG13, GNB1, and SLC6A3 (as shown in Figure 3B and Table 1).

### 3.4. Gene Ontology (GO) and KEGG Pathway Analyses

**Figure 4:**
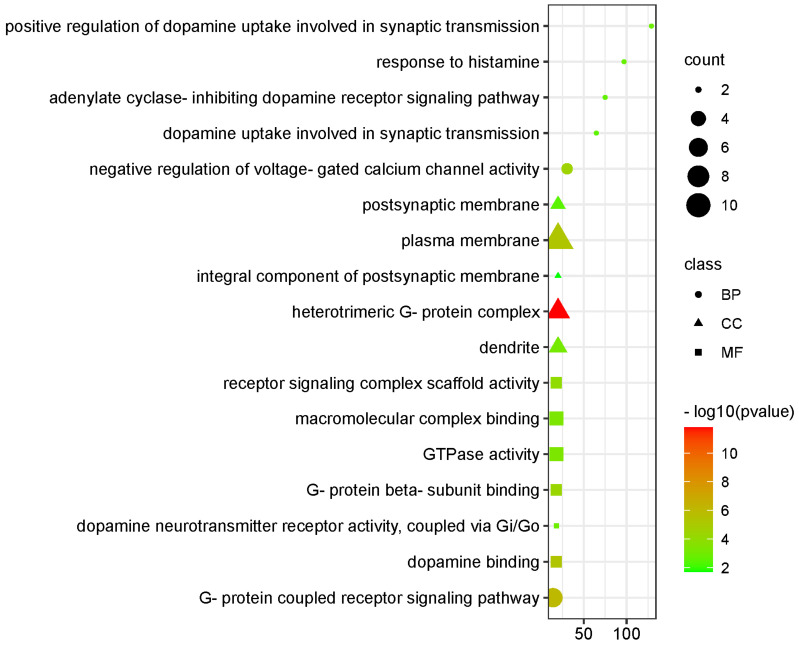
A bubble plot displaying the five most enriched GO, BP, CC, and MF terms of the top 10 hub genes identified by CytoHubba. GO, Gene Ontology; BP, biological process; CC, cellular component; and MF, molecular function.

An enrichment analysis of the top 10 targets for treating PD with quercetin was conducted using GO. A total of 49 items were obtained through the analysis, including 29 entries for biological processes, 9 for cell components, and 11 for molecular functions. The results showed that the top ten terms are arranged in descending order based on their *p*-values. The biological processes included the signaling pathways related to G-protein coupled receptors, the negative regulation of voltage-gated calcium channels, the positive regulation of dopamine uptake, the response to histamine, and adenylate cyclase inhibition. The cellular responses included the heterotrimeric G-protein complex, plasma membrane, dendrite, and an integral component of the postsynaptic membrane. The molecular functions were dopamine binding, G-protein beta-subunit binding, receptor signaling complex scaffold activity, macromolecular complex binding, and GTPase activity. The top five biological processes, cellular responses, and molecular functions are illustrated in Figure 4 based on the counts.

### 3.5. Signaling Pathways and Finding of Hub Signaling of QUE against PD

**Figure 5:**
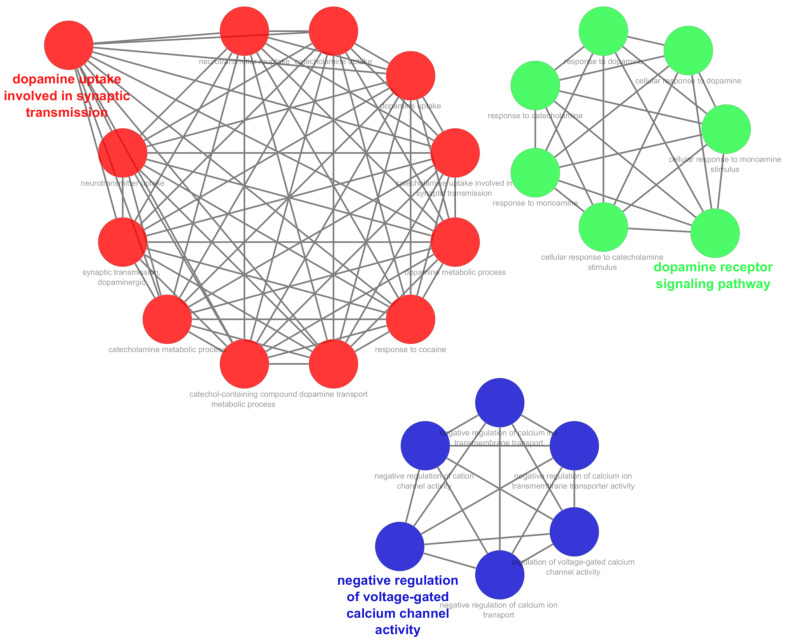
Cytoscape-ClueGo/CluePedia diagram presenting the protein–protein interactions of the top 10 hub genes identified by CytoHubba.

A KEGG pathway enrichment analysis was carried out (Figure 5), and all the pathways with a *p*-value of less than 0.05 were screened and ranked based on their *p*-value. The KEGG pathways primarily involved were those related to dopaminergic synapses, alcoholism, GABAergic synapses, morphine addiction, and circadian entrainment. Additionally, a ClueGo cluster analysis revealed that the KEGG-enriched pathways were primarily involved in dopamine uptake regulation for synaptic transmission, the dopamine receptor signaling pathway (Figure 5), and the negative regulation of voltage-gated calcium channel activity.

### 3.6. Component–Target–Pathway Network

**Figure 6:**
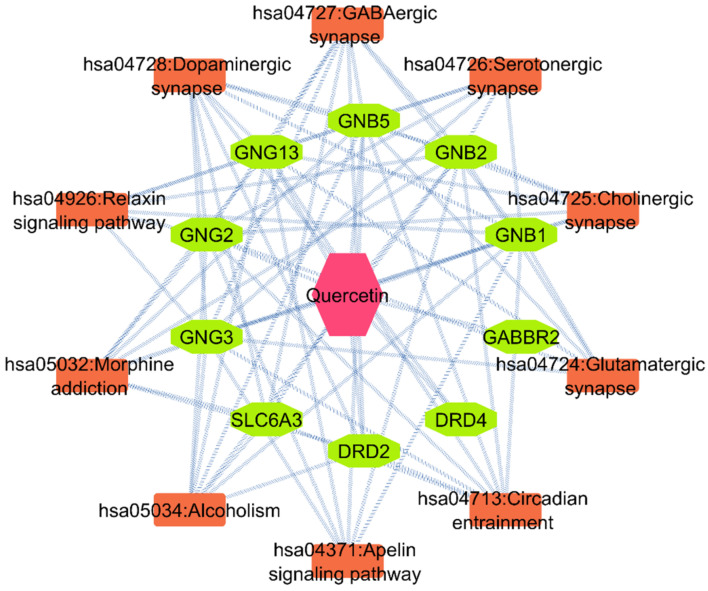
Component–target–pathway network of QUE in Parkinson’s disease. Pink represents Quercetin, green represents the targets, and orange represents the pathway.

To better understand the relationships between QUE, the targets, and the pathways, a comprehensive network was constructed using the top 10 pathways. This network identified 10 hub genes associated with QUE and other pathways (Figure 6). The topological parameters of the QUE treatment of the PD network were analyzed using the Network Analyzer in Cystoscope 3.6.0 to identify the core components and targets. Based on the results of the network analysis, DRD4 and DRD2 were predicted to be a significant genes due to their high degree of connections in the network. DRD4 and DRD2 were chosen for the molecular docking based on their involvement in KEGG, PPI, and the literature.

### 3.7. Molecular Docking

**Figure 7:**
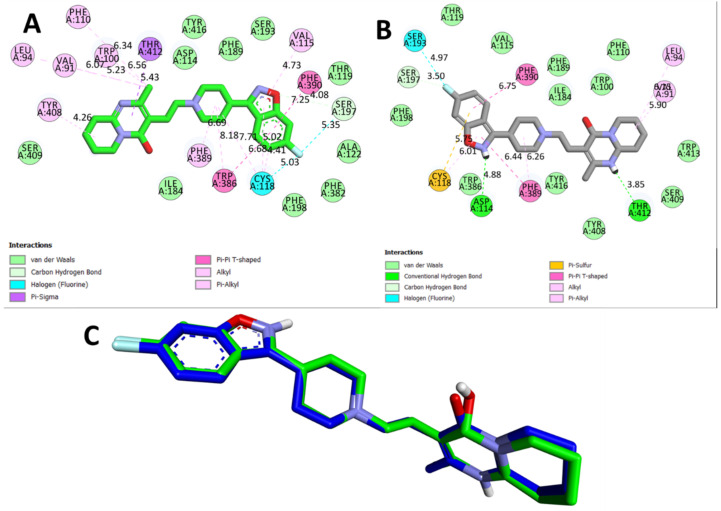
Different 2D bonding interactions between 6m4 and the native risperidone inhibitor. (**A**) Co-crystallized pose, (**B**) redocked pose, and (**C**) validation of the docking algorithm by redocking the native. Inhibitor risperidone on the target Dopamine D2 receptor (PDB ID:6CM4). Green: native crystallized pose of Risperidone; and blue: docked pose of risperidone.

**Figure 8:**
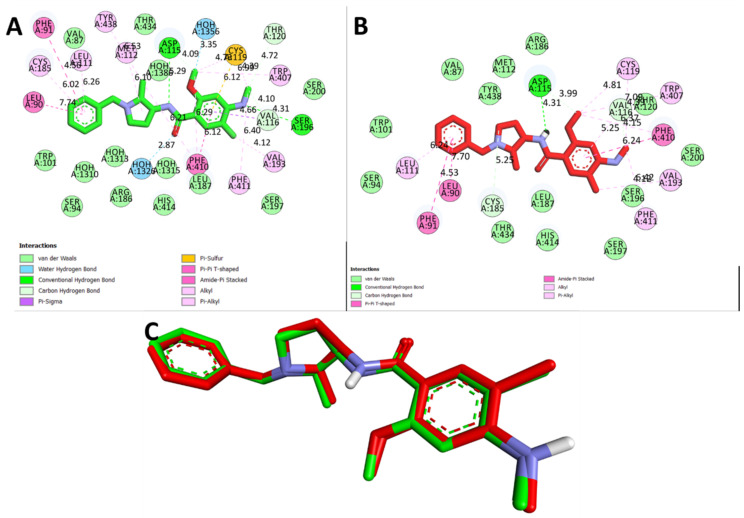
Different 2D bonding interactions between 5WIU and the native inhibitor nemonapride. (**A**) Co-crystallized pose, (**B**) redocked pose, and (**C**) validation of the docking algorithm by redocking the native. Inhibitor nemonapride on the target Dopamine D4 receptor (PDB ID:5WIU). Green: native crystallized pose of Risperidone; and red: docked pose of nemonapride.

The docking protocol was validated by redocking the co-crystallized ligands (risperidone for 6CM4 and nemonapride for 5WIU) into the active pocket of the binding site. The calculated root mean square deviation (RMSD) values between the redocked and co-crystallized poses were 0.9403 Å and 0.8854 Å for 6CM4 and 5WIU, respectively. These RMSD values demonstrate the efficiency and validity of the docking protocol (as shown in Figure 7 and Figure 8).

Table 2 provides the binding affinity (ΔG, kcal/mol), the involved amino acids, and the distances between them (in Å) for the three ligands.

**Figure 9:**
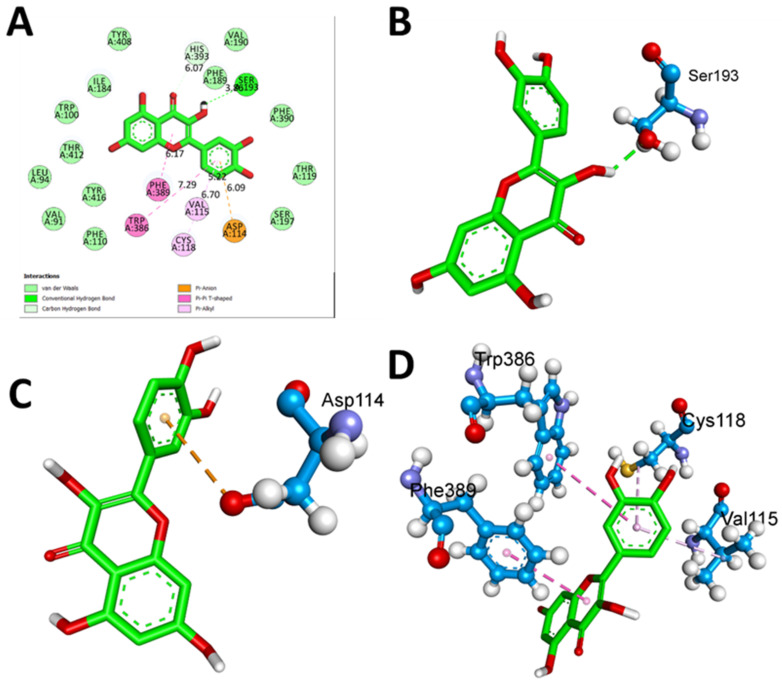
Two-dimensional and three-dimensional interactions of QUE with the D2 receptor (PDB ID:6CM4). (**A**) Two-dimensional view. (**B**) Three-dimensional interaction view of amino acid SER A:193 via a hydrogen bond. (**C**) Three-dimensional interaction view of amino acid SER A:193 via electrostatic interactions. (**D**) Three-dimensional interaction view of amino acids involved in hydrophobic bonds.

QUE displayed a strong binding affinity of −8.4 Kcal/mol with DRD2. The critical interface included multiple amino acids, resulting in various interactions. Significant hydrogen bond interactions were observed at SER A:193 at a distance of 3.86 Å (Figure 9B). Hydrophobic interactions were also prominent, with VAL A:115, CYS A:118, TRP A:386, PHE A:389, and HIS A:393 (Figure 9C), contributing distances ranging from 5.22 to 7.29 Å. Moreover, electrostatic interactions were observed with ASP A:114 at 6.09 Å (Figure 9D). This diverse array of interactions highlights the intricate nature of the QUE binding mechanism, which likely contributes to its potent binding affinity.

**Figure 10:**
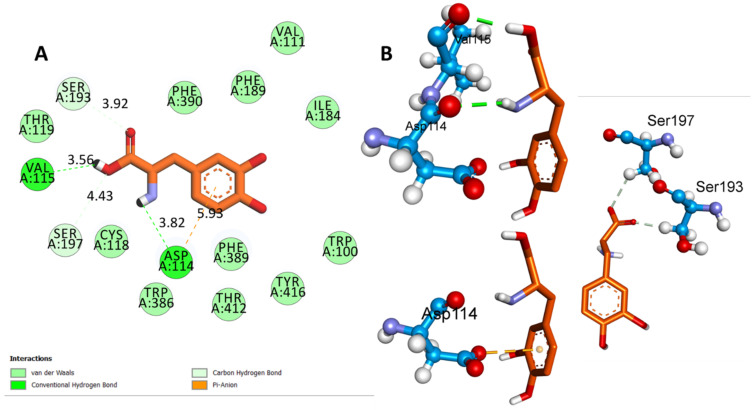
Depictions of the interactions between l-DOPA and the D2 receptor. (**A**) Two-dimensional interactions of l-DOPA with the D2 receptor (PDB ID:6CM4). (**B**)The 2D representation illustrates the interactions between l-DOPA and the D2 receptor (PDB ID:6CM4) via the hydrogen bond formed with Val 115, hydrophobic interactions with Ser 197 and Ser 193, and electrostatic interactions with Asp 114.

Figure 10A indicates that L-Dopa had a binding affinity of −6.4 Kcal/mol with DRD2. The interactions were mainly mediated by hydrogen bonding, involving ASP A:114 at a distance of 3.82 Å and VAL A:115 at 3.56 Å. Additionally, hydrophobic interactions were present between SER A:193 and SER A:197 at distances of 3.92 and 4.43 Å, respectively. Furthermore, electrostatic interactions were observed between ASP A114 and L-Dopa at a distance of 5.93 Å (Figure 10B). Despite having fewer interactions than QUE, the diverse range of interactions between L-Dopa and DRD2 led to its moderate binding affinity.

The ligand Risperidone, which was co-crystallized, exhibited a binding affinity of −8.0 Kcal/mol (Figure 7A,B). In this case, the interactions were primarily hydrophobic. Several amino acids, including VAL A at position 91, LEU A at position 94, TRP A at position 100, PHE A at position 110, TYR A at position 408, THR A at position 412, VAL A at position 115, PHE A at position 390, SER A at position 197, TRP A at position 386, PHE A at position 389, and CYS A at position 118, contributed to these hydrophobic interactions, ranging from 4.26 to 7.25 Å (Figure 8C). The absence of hydrogen bond interactions with the co-crystallized ligand indicated that hydrophobic forces played a crucial role in the stabilization of this complex.

**Figure 11:**
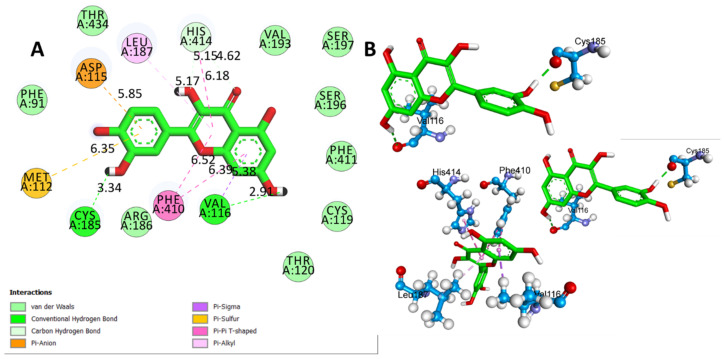
(**A**) Two-dimensional (2D) interactions of QUE with D4 receptor (PDB ID:5WIU) and (**B**) three-dimensional (3D) representations of L-DOPA hydrogen bond interactions with D4 receptor via Cys 185 and Val 116, hydrophobic interactions via Leu 187, His 414, Val 116, and Phe 410, and electrostatic interactions via Met 112 and Asp 115 (PDB ID:5WIU).

**Figure 12:**
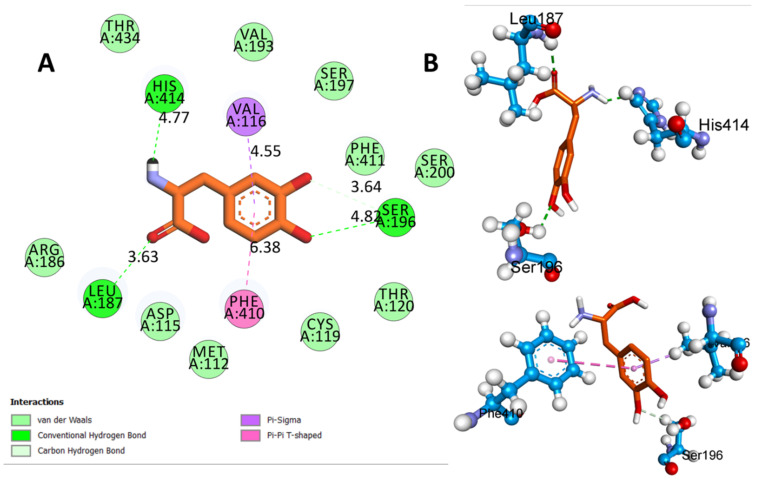
(**A**) Two-dimensional (2D) interactions of l-Dopa with the D4 receptor (PDB ID:5WIU) and (**B**) three-dimensional (3D) representation of L-DOPA with the D4 receptor (PDB ID:5WIU) via hydrogen bond interactions with Leu 187, Ser 196, and His 414, as well as hydrophobic interactions with Val 116, Phe 410, and Ser 196.

Table 2 presents the results of the molecular docking simulations that investigated the binding interactions between the two ligands, QUE and L-Dopa, and the D4 receptor (PDB:5WIU). QUE exhibited a remarkable binding affinity of −8.3 Kcal/mol to the D4 receptor (Figure 11A). Notable interactions included hydrogen bonds with VAL A:116 (2.91 Å) and CYS A:185 (3.34 Å), as well as hydrophobic interactions with LEU A:187 (5.17 Å), HIS A:414 (6.18 Å, 4.62 Å, and 5.15 Å), VAL A:116 (5.38 Å), and PHE A:410 (6.39 Å and 6.52 Å) (Figure 11B). Additionally, QUE displayed electrostatic interactions with MET A:112 (6.35 Å) and ASP A:115 (5.85 Å) (Figure 11C). L-Dopa exhibited a binding affinity of −6.2 Kcal/mol (Figure 12A) with DRD4. The interactions involved hydrogen bonds with LEU A:187 (3.63 Å), SER A:196 (4.82 Å), and HIS A:414 (4.77 Å), as well as hydrophobic interactions with VAL A:116 (6.38 Å), PHE A:410 (6.38 Å), and SER A:196 (3.82 Å) (Figure 12B).

### 3.8. Characterization of QNC Using Zeta Size Analysis

**Figure 13:**
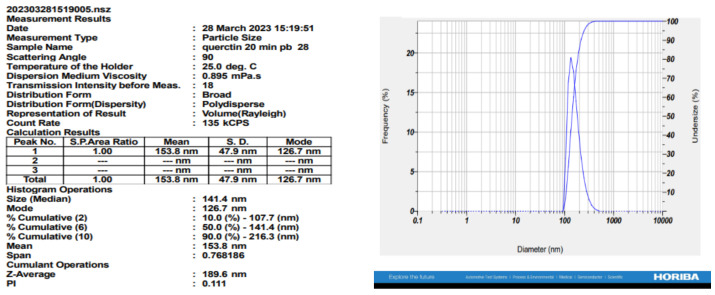
Zeta potential of QNC.

**Figure 14:**
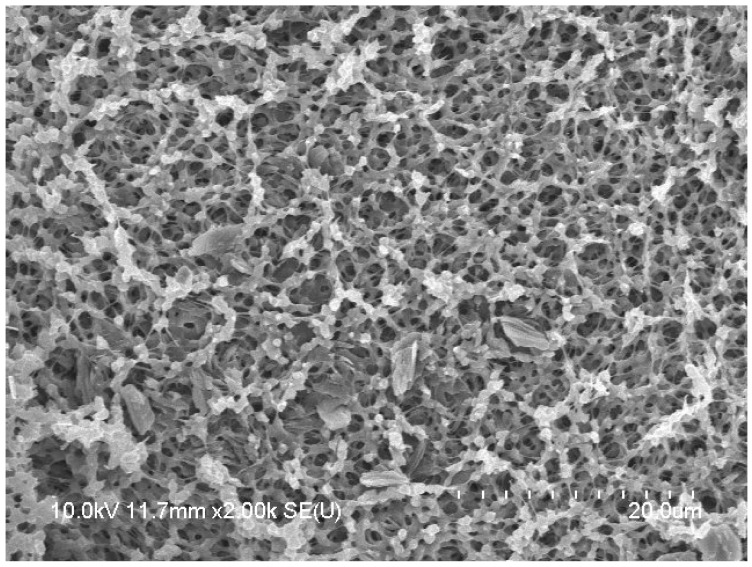
Scanning electron microscope photo of QNC.

The QNC displayed a particle size of 153.8 ± 47.9 nm and a polydispersity index of 0.111. The PDI, which ranges from 0 to 1, is a crucial factor affecting the in vivo performance of drugs. A PDI less than 0.3 signifies homogenous nanocrystals [39]. The nanocrystals showed a zeta potential of −44.0 mV, indicating that they were successfully formulated and stable, with an irregular and flake-type morphology (Figure 13 and Figure 14).

### 3.9. Effect of QNC on Motor Function in Rats

**Figure 15:**
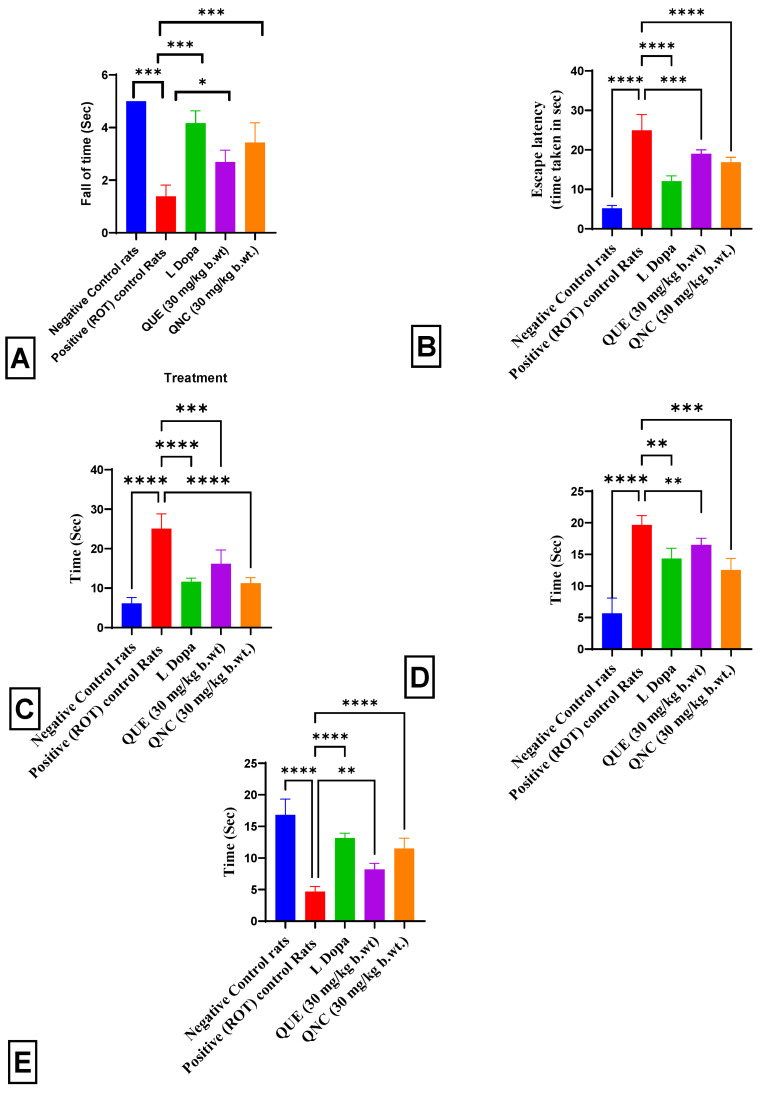
Graphical presentation of the results of motor function. (**A**) Rota Rod test. Positive (ROT) Control rats (*p* < 0.001 ***) vs. Negative Control rats; L-Dopa (*p* < 0.001 ***) treatment vs. Positive (ROT) Control rats; QNC (*p* < 0.001 ***) treatment vs. Positive (ROT) Control rats; and QUE (*p* < 0.05 *) treatment vs. Positive (ROT) Control rats. (**B**) Pole test. Positive (ROT) Control rats (*p* < 0.0001 ****) vs. Negative Control rats; L Dopa (*p* < 0.0001 ****) treatment vs. Positive (ROT) Control rats; QNC (*p* < 0.0001 ****) treatment vs. Positive (ROT) Control rats; and QUE (*p* < 0.001 ***) treatment vs. Positive (ROT) Control rats. (**C**) Stair test. Positive (ROT) Control rats (*p* < 0.0001 ****) vs. Negative Control rats; L Dopa (*p* < 0.0001 ****) treatment vs. Positive (ROT) Control rats; QNC (*p* < 0.0001 ****) treatment vs. Positive (ROT) Control rats; and QUE (*p* < 0.001 ***) treatment vs. Positive (ROT) Control rats. (**D**) Wood-walking test. Positive (ROT) Control rats (*p* < 0.0001 ****) vs. Negative Control rats; L-Dopa (*p* < 0.0001 ****) treatment vs. Positive (ROT) Control rats; QNC (*p* < 0.001 ***) treatment vs. Positive (ROT) Control rats; and QUE (*p* < 0.01 **) treatment vs. Positive (ROT) Control rats. (**E**) Wire-hanging test. Positive (ROT) Control rats (*p* < 0.0001 ****) vs. Negative Control rats; L-Dopa (*p* < 0.0001 ****) treatment vs. Positive (ROT) Control rats; QNC (*p* < 0.0001 ****) treatment vs. Positive (ROT) Control rats; and QUE (*p* < 0.01 **) treatment vs. Positive (ROT) Control rats.

The effects of QNC on the rotarod performance are depicted in (Figure 15A). A one-way ANOVA revealed significant differences in the rotarod performance among the groups [F(4, 25) = 49, *p* < 0.0001]. Locomotor dysfunction in the rotenone-treated rats was observed during the rotarod test. The retention time on the rotating rod for the group treated with rotenone decreased to 1.01 ± 0.01 s, which was significantly lower than the retention time observed for the normal rats on day 21. Conversely, the rats treated with the standard drug L-Dopa demonstrated a significant increase in their retention time on the rotating rod, measuring 4.275 ± 0.03 s, compared to the rats treated with rotenone. On the 21st day of the examination, the rats treated with QNC showed a statistically significant increase in their retention time (3.85 ± 0.045 s) compared to the rats treated with QUE and rotenone.

The pole-climbing test revealed neurological and motor system dysfunctions (Figure 15B). The impact of QNC on performance in the pole-climbing test is shown. A one-way ANOVA revealed significant differences in the escape latency among the treatment groups (F (4, 25) = 78, *p* < 0.0001). The escape latency was characterized by a restriction of the laddering period. The rotenone treatment group rats showed a significant increase in their escape latency time to 28.7 ± 1.76 s compared to the normal rats on the 21st day of the evaluation. In contrast, the L-Dopa treatment group rats showed a significant decrease in their escape latency time to 13 ± 0.67 s compared to the rotenone treatment group rats. However, the QNC treatment group rats showed a significantly decreased latency time of 16.5 ± 1.67 s compared to the QUE-treated and rotenone-treated rats on the 21st day of the evaluation.

The effects of QNC on skilled motor coordination using the stair test are presented in Figure 15C. A one-way ANOVA revealed significant differences in the time spent on the stairs between the treatment groups (F (4, 25) = 48, *p* < 0.0001). The time spent on the stairs was significantly increased in the rotenone-treated rats (24.3 ± 0.43 s) compared to the normal rats. At the same time, treatment with the standard drug L-Dopa resulted in a significant decrease (13 ± 0.67 s) compared to the rotenone-treated group. However, the QNC treatment resulted in a substantial decrease (10.6 ± 0.13 s) in the time spent on the stairs compared to both the QUE-treated and rotenone-treated rats on the 21st day of evaluation.

The results of the wood-walking test are presented in Figure 15D, demonstrating the effects of QNC on motor coordination. A one-way ANOVA revealed significant differences in the time spent walking on the wood sticks among the treatment groups (F (4, 25) = 53.29, *p* < 0.0001 *). The rotenone treatment group showed a significant decrease in the time spent on the wood sticks compared to the normal rats, with an average time of 18.3 ± 0.33 s. Similarly, the L-Dopa treatment group showed a significant decrease in the time spent on the wood sticks compared to the rotenone treatment group, with an average time of 14.3 ± 0.15 s. However, the QNC treatment group showed a significant decrease in the time spent on the wood sticks compared to the QUE and rotenone treatment groups, with an average time of 12.5 ± 0.15 s on the 21st day of the evaluation.

The effects of QNC on motor tone using the wire-hanging test are demonstrated in (Figure 15E). A one-way ANOVA revealed significant differences in the time spent on the stairs between the treatment groups (F (4, 25) = 58.98, *p* < 0.0001). The latency time for the rotenone-treated rats was 4.3 ± 0.13 s, significantly lower than that of the normal rats, while the L Dopa-treated rats had a significantly increased time spent on the wire of 14.6 ± 0.13 s. However, the QNC treatment significantly decreased the time spent to 13.5 ± 0.25 s compared to both the rotenone- and QUE-treated rats on the 21st day of the evaluation.

### 3.10. Effect of QNC on Tissue Biochemical Parameters

#### 3.10.1. Catalase (CAT) Levels

**Figure 16:**
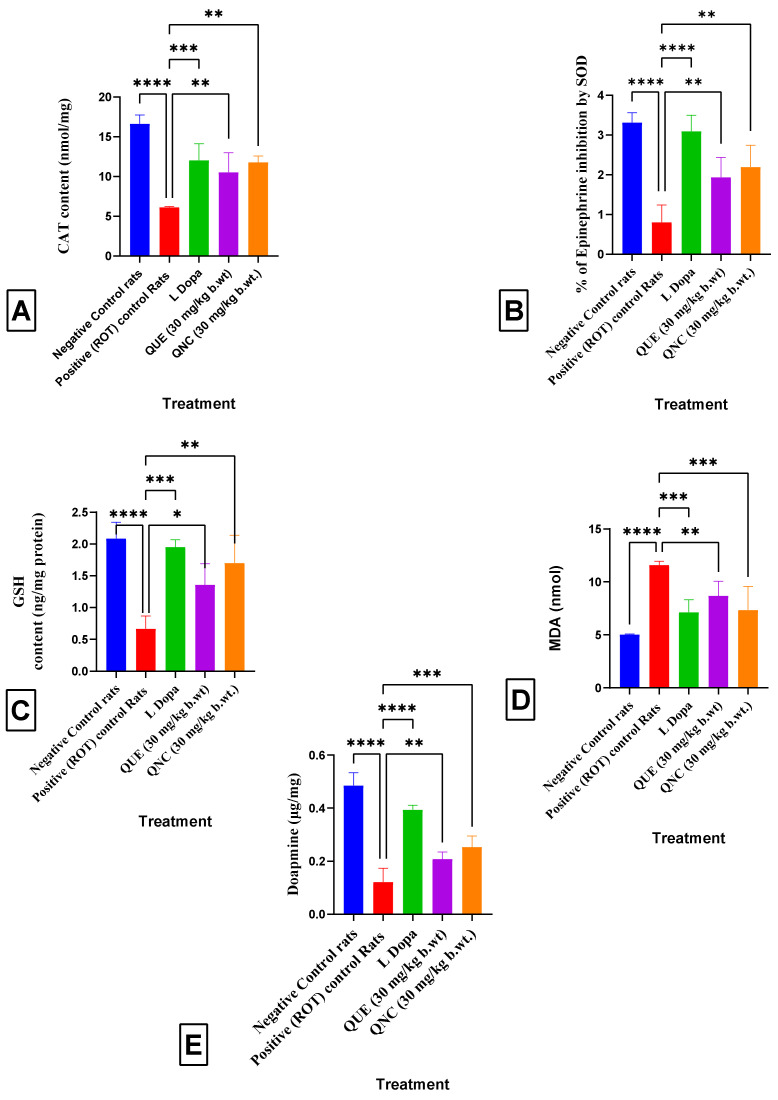
Graphical presentation of the results of brain biochemical parameters. (**A**) Catalase level. Positive (ROT) Control rats (*p* < 0.0001 ****) vs. Negative Control rats; L Dopa (*p* < 0.001 ***) treatment vs. Positive (ROT) Control rats; QNC (*p* < 0.01 **) treatment vs. Positive (ROT) Control rats; and QUE (*p* < 0.01 **) treatment vs. Positive (ROT) Control rats. (**B**) SOD level. Positive (ROT) Control rats (*p* < 0.0001 ****) vs. Negative Control rats; L-Dopa (*p* < 0.0001 ****) treatment vs. Positive (ROT) Control rats; QNC (*p* < 0.01 **) treatment vs. Positive (ROT) Control rats; and QUE (*p* < 0.01 **) treatment vs. Positive (ROT) Control rats. (**C**) GSH level. Positive (ROT) Control rats (*p* < 0.0001 ****, vs. Negative Control rats; L-Dopa (*p* < 0.001 ***) treatment vs. Positive (ROT) Control rats QNC (*p* < 0.01 **) treatment vs. Positive (ROT) Control rats; and QUE (*p* < 0.05 *) treatment vs. Positive (ROT) Control rats. (**D**) MDA level. Positive (ROT) Control rats (*p* < 0.0001 ****) vs. Negative Control rats; L Dopa (*p* < 0.001 ***) treatment vs. Positive (ROT) Control rats; QNC (*p* < 0.001 ***) treatment vs. Positive (ROT) Control rats; and QUE (*p* < 0.01 **) treatment vs. Positive (ROT) Control rats. (**E**) Dopamine level. Positive (ROT) Control rats (*p* < 0.0001 ****) vs. Negative Control rats; L-Dopa (*p* < 0.0001 ****) treatment vs. Positive (ROT) Control rats; QNC (*p* < 0.001 ***) treatment vs. Positive (ROT) Control rats; and QUE (*p* < 0.01 **) treatment vs. Positive (ROT) Control rats.

A one-way ANOVA revealed significant differences in the catalase levels between the treatment groups [F(4, 20) = 28, *p* < 0.0001]. The Dennett multiple comparison test showed that the brain CAT levels were significantly decreased in the rotenone treatment group (5.09 ± 0.43) compared to the normal rats (15.23 ± 0.13), while the CAT levels of the rats treated with the standard drug L-Dopa were significantly increased (12.6 ± 0.08) compared to the rotenone treatment rats. However, the QNC treatment significantly increased the brain CAT activity (11.5 ± 0.18) compared to the QUE rats (10.23 ± 0.08) and rotenone-treated rats on the 21st day of the evaluation. The administration of rotenone resulted in reduced CAT levels in the brain. However, when co-treated with QNC, there was a considerable increase in CAT levels. CAT has also been documented as a significant etiological component of Parkinson’s [49] (Figure 16A).

#### 3.10.2. Superoxide Dismutase (SOD) Levels

A one-way ANOVA revealed significant differences in the SOD levels among the treatment groups [F(4, 20) = 26.05, *p* < 0.0001]. The Dennett multiple comparison test showed that the brain SOD levels were significantly lower in the rotenone-treated rats (0.98 ± 0.08) compared to the normal rats (3.2 ± 0.50). In contrast, the L-Dopa treatment rats had significantly higher brain SOD levels (2.9 ± 0.2) compared to the rotenone-treated rats. However, the QNC treatment rats had significantly higher brain SOD levels (2.3 ± 0.46) compared to both the rotenone-treated and QUE-treated rats, and the difference was significant on the 21st day of the evaluation (Figure 16B).

#### 3.10.3. Glutathione (GSH) Levels

A one-way ANOVA revealed that there were significantly different levels of GSH among the treatment groups [F(4, 20) = 19, *p* < 0.0001]. The Dennett multiple comparison test revealed that the brain GSH levels were significantly reduced in the rotenone-treated rats (0.85 ± 0.002) compared to the normal rats (2.2 ± 0.050). In contrast, the L-Dopa treatment reversed the effect and significantly increased the brain GSH levels (1.9 ± 0.03) in the rats compared to the rotenone treatment (Figure 16C).

#### 3.10.4. Lipid Peroxidation (MDA) Levels

A one-way ANOVA revealed significant differences in the MDA levels between the treatment groups [F(4, 20) = 17.21, *p* < 0.0001]. Dennett’s multiple comparison tests revealed that the brain MDA levels were significantly higher in the rotenone-treated rats (10.2 ± 0.02) compared to the normal rats (4.6 ± 0.07), and treatment with the standard drug L-Dopa significantly decreased the brain MDA levels (7.1 ± 0.05) compared to the rotenone-treated rats. However, the QNC treatment significantly reduced the brain MDA levels (6.9 ± 0.06) compared to the QUE treatment and rotenone treatment on the 21st day of the evaluation (Figure 16D).

#### 3.10.5. Dopamine Levels

A one-way ANOVA showed significant differences in the dopamine levels among the treatment groups [F(4, 20) = 70, *p* < 0.0001]. Dennett’s multiple comparison test revealed that the brain dopamine levels were significantly decreased in the rotenone-treated rats (0.14 ± 0.002) compared to the normal rats (0.45 ± 0.007), while treatment with the standard drug L-Dopa considerably increased the brain dopamine levels to 0.39 ± 0.005 compared to the rotenone treatment. However, the QNC treatment increased the brain dopamine levels to 0.29 ± 0.06 in the rats, significantly higher than that in the QUE-treated rats (0.19 ± 0.024) and the rotenone treatment on the 21st day of the evaluation (Figure 16E).

### 3.11. Histological Results

**Figure 17:**
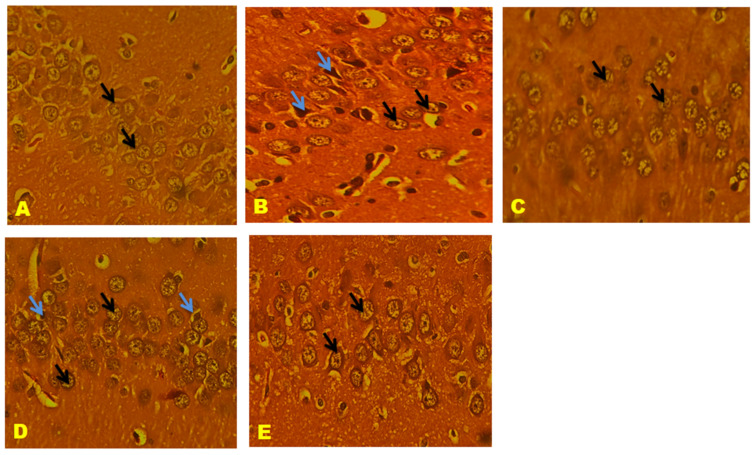
(**A**) Normal rat, (**B**) rotenone-induced Parkinson’s disease rat, (**C**) L-Dopa treatment + rotenone, (**D**) QUE treatment + rotenone, and (**E**) QNC treatment + rotenone. A section of CA2 stained with H&E was viewed at original magnification (×400). Neuronal cells with large vesicular nuclei and sparse cytoplasm (black arrow); (**B**) the CA2 region of rotenone-treated rats had increased degeneration of neuronal cells and appeared dark stained (blue arrow); (**E**) QNC rats showed that most neurons were similar to those of controls (**A**) and Standard (**C**) L-Dopa-treated rats (black arrow); and (**D**) QUE-only-treated rats showed few neuronal cells with prominent nuclei and cytoplasm.

**Figure 18:**
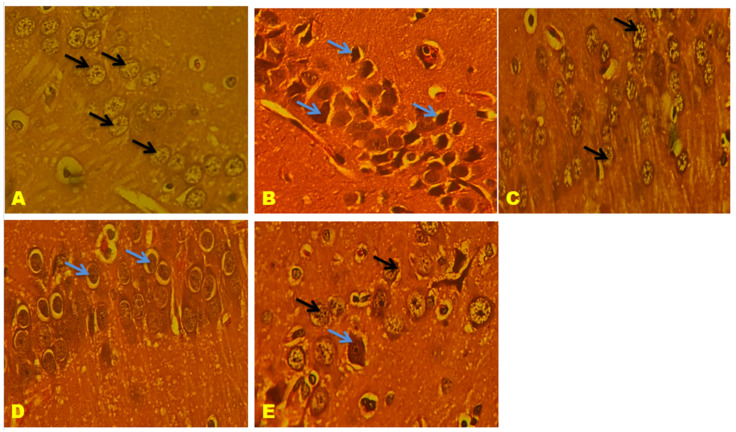
(**A**) Normal rat, (**B**) rotenone-induced Parkinson’s disease rat, (**C**) L-Dopa treatment + rotenone, (**D**) QUE treatment + rotenone, and (**E**) QNC treatment + rotenone. A section of CA1 stained with H&E was viewed at original magnification (×400). Neuronal cells with large vesicular nuclei and sparse cytoplasm ((black arrow); (**B**) the CA1 region of rotenone-treated rats had increased degenerated neuronal cells and appeared dark stained (blue arrow); (**E**) QNC rats showed that most neurons were similar to those of controls (**A**) and Standard (**C**) L Dopa-treated rats (black arrow), few dark stained dead cells; and (D) QUE-only-treated rats showed few neuronal cells with prominent nuclei, cytoplasm, and large dark-stained dead cells (blue arrow).

**Figure 19:**
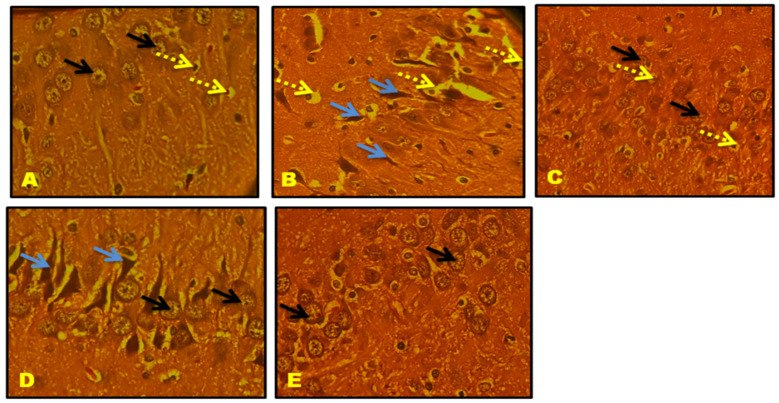
(**A**) Normal rat, (**B**) rotenone-induced Parkinson’s disease rat, (**C**) L-Dopa treatment + rotenone, (**D**) QUE treatment + rotenone, and (**E**) QNC treatment + rotenone. A section of CA3 stained with H&E was viewed at the original magnification (×400). Neuronal cells with large vesicular nuclei and sparse cytoplasm (black arrow) and astrocytes (dotted yellow arrow); (**B**) the CA3 region of rotenone-treated rats showed increased degeneration of neuronal cells (blue arrow), as well as perineuronal gaps inside, noticed hypertrophied astrocytes (dotted yellow); (**E**) QNC rats showed that most neurons were similar to those of controls and Standard L Dopa-treated rats (black arrow); and (**D**) QUE-only-treated rats showed few neuronal cells with prominent nuclei, cytoplasm, and elongated neurons.

**Figure 20:**
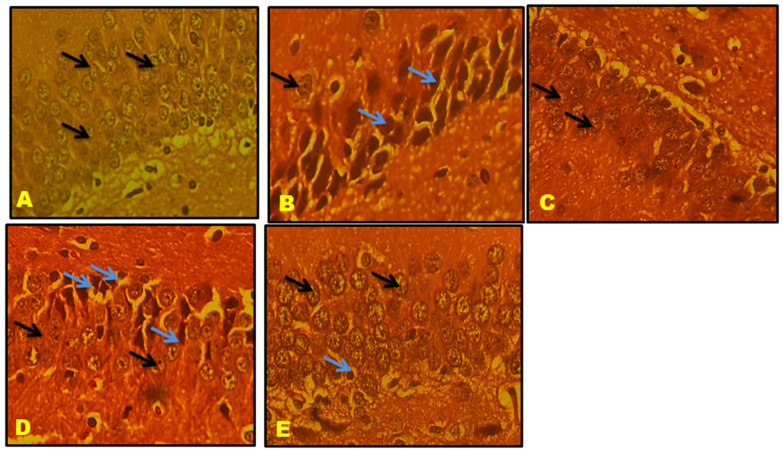
(**A**) Normal rat, (**B**) rotenone-induced Parkinson’s disease rat, (**C**) L-Dopa treatment + rotenone, (**D**) QUE treatment + rotenone, and (**E**) QNC treatment + rotenone. A section of the dentate gyrus (stained) stained with H&E was viewed at original magnification (×400). (**A**) The normal rat DG region shows dense glial cells with prominent nuclei (black arrows). (**B**) The DG region of rotenone-treated rats with damaged cells was sparsely arranged and their shapes were fuzzy. (**D**) QUE-treated rat DG shows glial cells with prominent nuclei in a dispersed manner (black arrow) and few damaged cells (blue arrow). (**E**) QNC-treated DG exhibited well-organized glial cells with prominent nuclei and no dead cells, similar to normal and L-Dopa rats.

**Figure 21:**
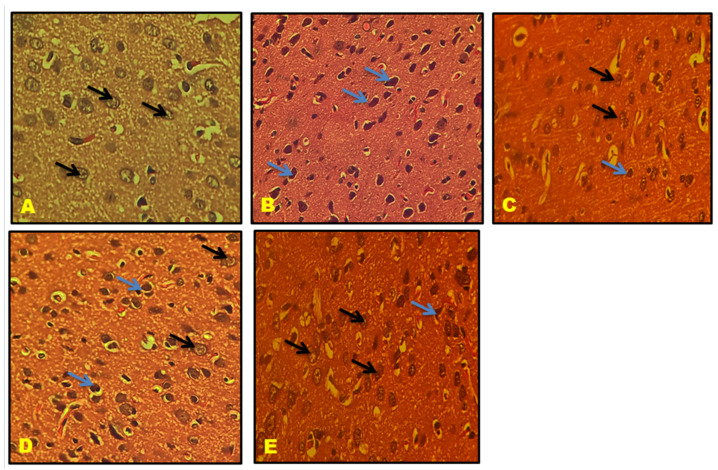
(**A**) Normal rat, (**B**) rotenone-induced Parkinson’s disease rat, (**C**) L-Dopa treatment + rotenone, (**D**) QUE treatment + rotenone, and (**E**) QNC treatment + rotenone. A section of the cerebral cortex stained with H&E was viewed at original magnification (×400). (**A**) Normal rat cortex shows vesicular rounded G Cells (black arrow); (**B**) degenerated shrunken neurons (blue arrow) with pyknotic nuclei; (**D**) appearance of prominent nuclei with rounded cells (black arrow) and the presence of few shrunken neurons (blue arrow); and QNC treatment rat cortex appeared nearly identical to normal rats and L-Dopa-treated rats.

**Figure 22:**
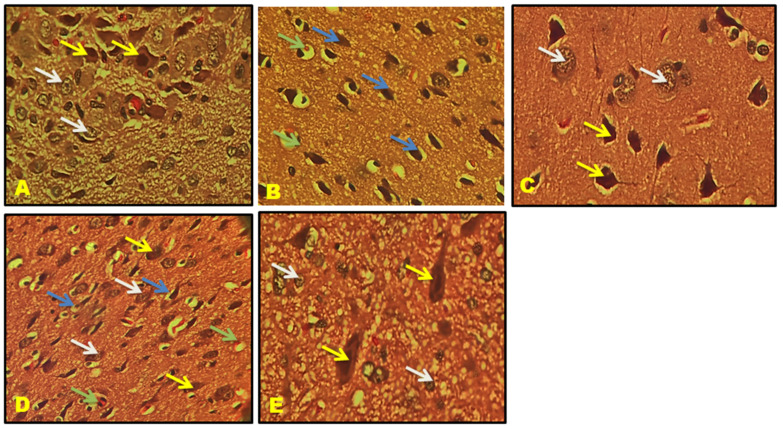
(**A**) Normal rat, (**B**) rotenone-induced Parkinson’s disease rat, (**C**) L-Dopa treatment + rotenone, (**D**) QUE treatment + rotenone, and (**E**) QNC treatment + rotenone. Sections of the SN stained with H&E were viewed at the original magnification (×400). (**A**) Normal rats showed large neurons and prominent nuclei (yellow arrows). Rounded neurons with nuclei (white arrows). (**B**) The SN in rotenone-treated rats exhibited intense neuronal loss and loss of rounded nuclei. Neurons were deeply stained, with the disappearance of nuclei and cytoplasmic inclusions of Lewy bodies (green arrow). (**D**). QUE-treated rats show prominent rounded neurons (yellow arrow), still appearance of necrotic cells (blue arrow), large neurons (white arrow), and few Lewy bodies (green arrow). (**E**) QNC rats showed that most neurons were similar to average and standard L-Dopa treatment (**C**), loss of Lewy bodies, and reappearance of round neurons.

CA1, CA2, CA3, and the Dentate gyrus (DG) of the hippocampal neuronal cells displayed large vesicular nuclei and sparse cytoplasm. The rotenone-treated cells in CA1, CA2, CA3, and DG exhibited an increased degeneration of neuronal cells and appeared dark stained, while the QNC treatment reduced neuronal cell damage compared to the QUE treatment. The cerebral cortex displayed vesicular rounded G cells, while rotenone caused shrunken neurons with pyknotic nuclei. The QNC treatment of the rat cortex appeared similar to that of the normal rats and L-Dopa-treated rats compared to the QUE treatment. In the substantia nigra region, rounded neurons with nuclei were observed, but the rotenone treatment resulted in the disappearance of nuclei and cytoplasmic inclusions of Lewy bodies. The QNC treatment showed a good degree of restoration of striatal neurons and the disappearance of Lewy bodies, which mostly had a near-normal appearance (Figure 17, Figure 18, Figure 19, Figure 20, Figure 21 and Figure 22).

## 4. Discussion

Recent years have seen significant attention given to natural products [50]. The network pharmacology method provides valuable insights into the complex interactions between medicines and their targets and their potential mechanisms of action [51,52]. However, discovering new drugs from plant sources presents methodological challenges [53]. The lack of ADME properties in newly discovered drugs, combined with the high cost of research, further complicates the drug discovery process [54]. Therefore, ADME-based screening is highly valuable for the development of pharmaceuticals [55]. Developing novel therapeutic strategies in response to the escalating neurodegenerative disease epidemic and the inadequate effectiveness of current FDA-approved medications is crucial. In this study, a network pharmacology approach was employed to investigate the mechanisms underlying the effects of QUE. The therapeutic targets and their signaling pathways were explored using PPI network construction and a pathway enrichment analysis. Reliable results were achieved using various target identification methods and integrating data from several online databases. The interactions between QUE and its targets were validated using molecular docking. The most probable targetmarks for QUE in Parkinson’s disease are twenty-one signaling pathways and the top ten proteins. These findings could improve our understanding of how QUE affects the treatment of neurodegenerative diseases, thus providing valuable information for future research on its use as a medical therapy for Parkinson’s disease.

The potential therapeutic use of QUE for Parkinson’s disease has been suggested, and the dopamine receptor signaling pathway and dopamine uptake in synaptic transmission mediate its effects. Other active small molecules have shown similar therapeutic mechanisms [56]. The therapeutic efficacy of QUE has been promising in rodent models of Parkinson’s disease [57], Alzheimer’s disease [58], and amyotrophic lateral sclerosis [59]. Previous studies have indicated that the dopamine uptake mechanism may have potential as a treatment. Exploring potential drug candidates and their underlying molecular mechanisms is crucial for drug research. The mechanism behind QUE’s effects will be discussed in the next section.

We investigated the QUE-related PD genes through the combination of a bioinformatics analysis and experimental verification. Our findings led to the identification of 120 genes associated with PD pathology and QUE treatment. We conducted PPI and functional enrichment analyses to gain further insights into their functions. Our results revealed that hub genes, such as DRD2, DRD4, DBH, and NTRK3, play critical roles in the biological pathway of QUE treatment. We also found that the G-protein-coupled receptor signaling pathway, the negative regulation of voltage-gated calcium channel activity, and the dopamine uptake involved in synaptic transmission may also be involved in QUE treatment. After analyzing the 120 genes, we selected DRD2 and DRD4 for experimental validation. Our results showed that both genes were differentially expressed in the GSE42966 dataset and were significantly changed after QUE treatment, indicating that they could serve as potential targets in PD and QUE treatment progression.

Two potential targets for the treatment of Parkinson’s disease (PD) are the dopamine D2 receptor (DRD2) and dopamine D4 receptor (DRD4). These receptors are expressed in the midbrain dopaminergic neurons, medium spiny neurons of the striatum, and various neuronal populations in the cerebral cortex [60]. The activation of these receptors can modulate dopaminergic signaling and potentially alleviate motor symptoms in PD.

Several studies have explored the parts played by DRD2 and DRD4 in PD treatment, and a meta-analysis has uncovered a significant association between the DRD4 rs2134655 polymorphism and PD, pointing to the possibility that genetic variations in DRD4 may have a hand in the disease’s development. Furthermore, research has demonstrated that Pramipexole, a dopamine agonist used to treat PD, boosts the DRD2 and DRD4 levels in the rat striatum [61]. This increase in expression may contribute to the drug’s therapeutic effects in PD patients.

In summary, DRD2 and DRD4 are significant in PD treatment. The activation of these receptors can regulate dopaminergic signaling, trigger autophagy, and potentially alleviate motor symptoms in PD. Nevertheless, more research is needed to completely comprehend the intricate mechanisms underlying the therapeutic effects of DRD2 and DRD4 in PD.

The molecular docking studies revealed that QUE had a binding affinity of −8.4 Kcal/mol, while the standard L-Dopa displayed a binding affinity of −6.4 Kcal/mol. This indicated that QUE had a better binding relationship than the standard drug L-Dopa. Akash Rathore et al.’s study also identified the critical structural amino acid residues of the D2 receptor as Asp114, Trp386, and Phe390. Interestingly, QUE interacted with two vital amino acids, Asp114 via electrostatic interactions and Trp386 via hydrophobic interactions, while the standard L-dopa interacted only with Asp114 via both hydrogen and electrostatic bond interactions [62]. Therefore, the top two ranked targets, DRD2 and DRD4, were evaluated using in vivo studies to determine the dopamine levels.

The utilization of QUE in pharmacology is limited due to its low oral bioavailability (less than 2%), poor permeability across the blood–brain barrier, and hydrophobic characteristics [63]. Nanocrystalline technology, with its increased surface area and ability to produce nanosized particles, has become a popular approach in drug delivery systems. This approach can improve efficiency by increasing bioavailability, allowing for dosage reduction, and enhancing efficacy [64]. In this study, QUE nanocrystals (QNC) were synthesized through a wet media milling method, and the Noyes–Whitney equation was used to show that reducing the particle size and increasing the particle surface area can increase bioavailability [65].

The current study found that administering rotenone to rats caused a significant decline in their motor coordination and muscle tone, as shown by the results of several tests, including the rota rod, pole, stair, wood-walking, and wire-hanging tests (Figure 15). These findings are consistent with those of Khalil et al. (2015) [66], who also observed neurodegeneration and the onset of Parkinson’s disease following rotenone administration at the same frequency. At the same time, previous research has suggested that QUE possesses antioxidant properties [67,68]. The therapeutic potential of QNC in mitigating rotenone-induced motor deficits in PD rats has not yet been investigated. However, the current study found that QNC administration improved the motor function in rats with rotenone-induced Parkinson’s disease compared to treatment with QUE.

Several investigations have shown that oxidative stress plays a crucial role in degenerating dopaminergic neurons [69]. The results of these studies indicated that rotenone administration led to increased oxidative damage in the rat brain, characterized by the decreased activity of antioxidant enzymes such as SOD, GSH, and CAT, along with elevated levels of MDA. These findings are consistent with those of a previous study conducted by Altharawi et al. [33]. Interestingly, pretreatment with QNC resulted in a significant decrease in MDA and an increase in the brain catalase, GSH, and SOD contents, similar to that observed in the L-Dopa group when compared to the QUE group.

Depleting neurotransmitters, particularly dopamine, is the primary cause of oxidative damage. Dopamine’s role is crucial in controlling movement and signal transmission [70]. The current study found that rotenone administration led to a significant reduction in dopamine levels and changes in its metabolites. These findings are consistent with previous studies by Hamed (2021) [71] and Zhao et al. (2021) [72]. The administration of QNC restored the brain dopamine levels to normal in the rats with rotenone-induced Parkinson’s disease, compared to QUE therapy. Previous histological investigations have shown significant neuronal death in the cortex, hippocampus, and substantia nigra of rats with rotenone-induced Parkinson’s disease [40]. However, the simultaneous administration of QNC significantly reduced neuronal cell death compared to treatment with QUE.

Based on these results, treatment with QNC significantly improved motor function, increased antioxidant enzyme activities, reduced the oxidative enzymes in brain homogenates, and decreased neuronal death compared to QUE treatment in PD rats. According to previous studies, QNC has been found to exhibit increased free radical scavenging in the direction of coarse QUE [73]. The current study also found that increased particle bioavailability improved the neuroprotective effect of QUE in its nanocrystal form.

## 5. Conclusions

Based on network pharmacology and in vivo experiments, we found that QUE has anti-Parkinson effects by increasing the sensitivity of dopamine to the D2 and D4 receptors. Our study suggested that QNC can attenuate the behavioral, biochemical, neurochemical, and histopathological alterations caused by rotenone-induced oxidative stress. This can be attributed to QNC’s antioxidant ability, which reduced the oxidative damage in rat brains compared to unformulated QUE treatment. Therefore, QNC can be a potential treatment for PD as a herbal phytoconstituent and serve as a basis for future clinical studies.

## Figures and Tables

**Table 1:** Top 10 hub nodes selected using the MCC method in Cytohubba.

Rank	Abbreviation	Full Form	Description
1	DRD2	Dopamine Receptor D2	A gene encoding a dopamine receptor subtype implicated in various neurological and psychiatric conditions.
2	DRD4	Dopamine Receptor D3	Another dopamine receptor subtype involved in neuropsychiatric disorders.
3	DBH	Dopamine Beta-Hydroxylase	It is involved in the conversion of dopamine into norepinephrine and has relevance to various neurological and psychiatric conditions.
4	NTRK3	Neurotrophic Tyrosine Kinase Receptor Type 3	Encodes a receptor for neurotrophins and plays a role in neuronal development and function.
5	CDH1	Cadherin-1	Encodes a protein involved in cell adhesion and is associated with various cancers, particularly gastric cancer.
6	GFRA1	GDNF Family Receptor Alpha 1	A receptor for the glial cell line-derived neurotrophic factor (GDNF) that plays a role in neuronal development and maintenance.
7	BDNF	Brain-Derived Neurotrophic Factor	A gene encodes a protein that supports the survival and growth of neurons. It is important in neurodevelopment and mental health.
8	CCND1	Cyclin D1	Encodes a protein involved in cell cycle regulation and is frequently overexpressed in cancer.
9	CREBBP	CREB-Binding Protein	It is important for gene regulation and is associated with Rubinstein–Taybi syndrome and other conditions.
10	RET	Ret Proto-Oncogene	Associated with developing multiple endocrine neoplasia type 2 (MEN2) and other cancers.

**Table 2:** Binding energies and interaction details of QUE, L-Dopa, and co-crystallized ligand with D4 receptor (PDB:6CM4).

Ligands	Target	Binding Affinity, ΔG (Kcal/mol)	Amino Acids Involved and Distance (Å)		
			Hydrogen Bond Interactions	Hydrophobic Interactions	Electrostatic Interactions
QUE	D2 receptor (PDB:6CM4)	−8.4	SER A:193 (3.86)	VAL A:115 (5.22), CYS A:118 (6.70), TRP A:386 (7.29), PHE A:389 (6.17), HIS A:393 (6.07)	ASP A:114 (6.09)
L-Dopa		−6.4	ASP A:114 (3.82), VAL A:115 (3.56)	SER A:193 (3.92), SER A:197 (4.43)	ASP A:114 (5.93)
Co-Crystallized Ligand (Risperidone)		−8.0	-	VAL A:91 (5.23), LEU A:94 (6.07), TRP A:100 (6.56), PHE A:110 (6.34), TYR A:408 (4.26), THR A:412 (5.43), VAL A:115 (4.73), PHE A:390 (7.25), SER A:197 (5.03), TRP A:386 (5.02), PHE A:389 (6.69), CYS A:118 (5.03, 4.41, 5.02, 6.68)	-
QUE	D4 receptor (PDB:5WIU)	−8.3	VAL A:116 (2.91), CYS A:185 (3.34)	LEU A:187 (5.17), HIS A:414 (6.18, 4.62, 5.15), VAL A:116 (5.38), PHE A:410 (6.39, 6.52)	MET A:112 (6.35), ASP A:115 (5.85)
L-Dopa		−6.2	LEU A:187 (3.63), SER A:196 (4.82), HIS A:414 (4.77)	VAL A:116 (6.38), PHE A:410 (6.38), SER A:196 (3.82)	-
Co-Crystallized Ligand (Nemonapride)		−8.7	ASP A:115 (4.31)	LEU A:90 (7.70), PHE A:91 (4.53), LEU A:111 (6.24), ASP A:115 (3.99), CYS A:119 (4.81, 7.09), TRP A:407 (4.39), PHE A:410 (5.25, 6.24), VAL A:193 (4.12), PHE A:411 (6.42)	-

## Data Availability

We analyzed existing, publicly available data. The accession numbers for the datasets are listed in the key resources table. Any additional information required to reanalyze the data reported in this paper is available from the lead author upon request.
